# Modulation of LXR signaling altered the dynamic activity of human colon adenocarcinoma cancer stem cells *in vitro*

**DOI:** 10.1186/s12935-021-01803-4

**Published:** 2021-02-10

**Authors:** Hassan Dianat-Moghadam, Mostafa Khalili, Mohsen Keshavarz, Mehdi Azizi, Hamed Hamishehkar, Reza Rahbarghazi, Mohammad Nouri

**Affiliations:** 1 Department of Medical Biotechnology, Faculty of Advanced Medical Sciences, Tabriz University of Medical Sciences, Tabriz, Iran; 2 Stem Cell Research Center, Tabriz University of Medical Sciences, Tabriz, Iran; 3 The Persian Gulf Tropical Medicine Research Center, The Persian Gulf Biomedical Sciences Research Institute, Bushehr University of Medical Sciences, Bushehr, Iran; 4 Drug Applied Research Center, Tabriz University of Medical Sciences, Tabriz, Iran; 5 Department of Applied Cell Sciences, Faculty of Advanced Medical Sciences, Tabriz University of Medical Sciences, Tabriz, Iran

**Keywords:** Colorectal cancer, Cancer stem cell, LXR, ATP-binding cassette, Warburg effect, Clonogenicity, Metastasis

## Abstract

**Background:**

The expansion and metastasis of colorectal cancers are closely associated with the dynamic growth of cancer stem cells (CSCs). This study aimed to explore the possible effect of LXR (a regulator of glycolysis and lipid hemostasis) in the tumorgenicity of human colorectal CD133 cells.

**Methods:**

Human HT-29 CD133^+^ cells were enriched by MACS and incubated with LXR agonist (T0901317) and antagonist (SR9243) for 72 h. Cell survival was evaluated using MTT assay and flow cytometric analysis of Annexin-V. The proliferation rate was measured by monitoring Ki-67 positive cells using IF imaging. The modulation of LXR was studied by monitoring the activity of all factors related to ABC transporters using real-time PCR assay and western blotting. Protein levels of metabolic enzymes such as PFKFB3, GSK3β, FASN, and SCD were also investigated upon treatment of CSCs with LXR modulators. The migration of CSCs was monitored after being exposed to LXR agonist using scratch and Transwell insert assays. The efflux capacity was measured using hypo-osmotic conditions. The intracellular content of reactive oxygen species was studied by DCFH-DA staining.

**Results:**

Data showed incubation of CSCs with T0901317 and SR9243 reduced the viability of CD133 cells in a dose-dependent manner compared to the control group. The activation of LXR up-regulated the expression and protein levels of ABC transporters (ABCA1, ABCG5, and ABCG8) compared to the non-treated cells (p < 0.05). Despite these effects, LXR activation suppressed the proliferation, clonogenicity, and migration of CD133 cells, and increased hypo-osmotic fragility (p < 0.05). We also showed that SR9243 inhibited the proliferation and clonogenicity of CD133 cells through down-regulating metabolic enzymes PFKFB3, GSK3β, FASN, and SCD as compared with the control cells (p < 0.05). Intracellular ROS levels were increased after the inhibition of LXR by SR9243 (p < 0.05).

Calling attention, both T0901317 and SR9243 compounds induced apoptotic changes in cancer stem cells (p < 0.05).

**Conclusions:**

The regulation of LXR activity can be considered as a selective targeting of survival, metabolism, and migration in CSCs to control the tumorigenesis and metastasis in patients with advanced colorectal cancers.

## Background

Colorectal cancer (CRC) is one of the most common malignancies in the world [1], and despite current developments in treatment and screening methods, today nearly 50 % of patients with advanced CRC experience tumor relapse and metastasis [2]. The failure of CRC therapy could be explained by the presence of the rare population of CSCs which causes drug resistance, tumorigenesis, recurrence, and metastasis [3]. These cells could need on-demand energy for bioactivity and differentiation during the cancer stroma [4]. It has been shown that CRC development is affected by a high-fat diet or CHOL consumption [1, 5]. CHOL is a sterol that has proliferative effects on cells *via* supporting the cell membrane integrity and CHOL-rich membranes (or lipid rafts) formation [5]. For example, the CHOL content acts as putative mitogen through SREBP-2 (sterol regulatory element-binding protein) expression that promotes the proliferation and tumorigenesis of intestinal stem cells [6]. It has also shown that the lipid raft membrane-localized matrix metalloproteinase-2 induces the migration of stem cells through degrading the ECM and activation of matrix metalloproteinase-9 [7].

In normal cells, ABCs had the potential to efflux the intracellular CHOL to the ECM. Notably, it has been elucidated that these proteins have been suppressed during pathological cancer conditions [8]. LXRs are touted as the members of the nuclear receptor and form an activated heterodimer with RXRs to binds to LXR-responsive elements in DNA. LXRs could modify the transcription of target genes participating in tumor cell survival, metabolism, and migration [9]. LXR regulates elevated colon cellular CHOL by disrupting the CHOL synthesis-related effector such as SREBP, and upregulation of the ABC efflux pumps expression [10]. While activation of LXRs signaling is well studied in various types of somatic cancer cells, the critical role of LXRs has not been addressed in the dynamic growth of CSCs. In addition to the vital role of LXR-related signaling in CSCs activity, the proliferation of these cells is closely related to glycolytic pathway-dependent for ATP generation.

Calling attention, increased aerobic glycolysis (termed also Warburg effect) is touted as *de novo* lipogenesis which sustains cell proliferation and protects CSCs against stresses [11]. Among the several enzymes, the PFKFB3, and GSK3β are critical key enzymes involved in the control of glycolysis [12–14]. The surplus metabolites produced by the aerobic glycolysis are integrated into lipogenesis and thus this phenomenon increases the expression of lipid metabolism enzymes [e.g., FASN and SCD1] to induce endogenous production of lipids in tumor cells [12–14]. Moreover, inactivation of LXRs can inhibit the Warburg effect and lipogenesis, promoting apoptosis in somatic tumor cells [12]. To the best of our knowledge, there are few reports regarding the role of LXR-derived signaling pathways in the survival and dynamic growth of CSCs.

Commensurate with these descriptions, we investigated the effect of LXR activation on the growth, proliferation, and migration of HT-29-isolated CSCs *in vitro*. We further studied the inactivation of LXR on CR-CSCs glycolysis and lipid metabolism.

## Materials and methods

### Cell culture

HT-29 cell line was purchased from the National Cell Bank of Iran (NCBI; Pasteur Institute, Tehran), and cultured in RPMI 1640 (Gibco, USA) medium supplemented with 10% v/v FBS (Gibco, USA), 100 IU/ml Penicillin, and 100 µg/ml Streptomycin (Gibco, USA). To expand the cells, the culture flasks were maintained at a humidified atmosphere of 95% air and 5% CO_2_ at 37 °C. Cells were sub-cultured at 70–80% confluence using 0.25% Trypsin-EDTA (Gibco). In this study, HT-29 cells at passages 3–6 were used for subsequent analyses.

### Chemical and preparation procedure

In this study, we used T0901317 (TOCRIS) as an LXR agonist and SR9243 (TOCRIS, china) as a selective LXR antagonist to stimulate and inhibit LXR signaling pathways in the isolated CSCs. The chemicals were dissolved in DMSO (Merck, Germany) to prepare 0.2 M (50 mg/ml) stock solutions.

### Isolation of CSCs using MACS

In the current experiment, CSCs were enriched based on stemness surface marker CD133 using MSACS according to the manufacturer’s instruction. In short, the adherent HT-29 cells were detached using 0.25 % trypsin-EDTA, washed twice with PBS. The non-specific binding sites were blocked with 1 % BSA (Sigma-Aldrich). To isolate CD133^+^ cells, cells (42 × 10^6^) were incubated with 120 µl of microbeads against CD133 (Miltenyi Biotec., Germany) at 4 °C for 20 minutes on a rotator. Thereafter, cells were washed with PBS containing 1 % BSA and passed through LS columns (Miltenyi Biotec., Germany). Soon after enrichment, CD133^+^ cells were collected as primary CSCs for subsequent studies.

### Characterization of isolated CSCs by flow cytometry

To confirm the purity of enriched CSCs, we performed flow cytometric analysis of CD133. The samples were washed twice with PBS and incubated with 10 µl of the FITC-conjugated anti-human CD133 antibody (Clone: AC133; Cat No. 130-113-673; Miltenyi Biotec) at 4 °C for 30 minutes. Then, cells were washed twice with PBS and fixed with 4 % PFA solution. Cells were analyzed using a FACSCalibur flow cytometer (BD Bioscience, USA) and FlowJo software (version 7.6.1). The purity of cells was compared with control HT-29 cells before the MACS procedure.

### Cell viability assay

The effect of T0901317 and SR9243 were determined on CSC viability by a colorimetric assay using MTT (Sigma-Aldrich, USA). Briefly, 1 × 10^4^ CSCs resuspended in culture medium enriched with 2 % FBS, transferred onto each well of 96-well plates, and allowed reach 70–80% confluence. After 24 h, cells were exposed to various concentrations of T0901317 (5–200 µM) or SR9243 (25–200 nM) for 24, 48, and 72 h. After completion of the treatment protocol, 20 µl of MTT (5 mg/ml) solution was added to each well and incubated for the next 4 h. Thereafter, the culture medium was removed and replaced with 200 µl DMSO solution. Finally, the absorbance value was measured at 630 nm using a microplate reader (BioTeK Instruments, USA). Cell viability (IC_50_) was determined for each agent by calculating the slope and intercept of different concentrations.

### Real‐time PCR assay

Total RNA was extracted using RNA X plus solution (CinnaGen, Iran) according to the manufacturer’s procedure. The extracted RNA was reverse-transcribed into cDNA by a reverse transcription Kit (Bioneer, Korea). Real-time PCR analysis was performed using QuantiTect SYRB Green dye (TaKaRa, Japan) and Corbett Rotor-Gene™ 6000 HRM system. Primer sequences were listed in Table 1. The expression levels of each gene were analyzed by Pfafl methods with normalization to the housekeeping gene, GAPDH. Three sets of experiments were conducted.

**Table 1:** Primer list

Gene	Forward primer	Reverse primer	Tm (°C)
*ABCA1*	TTCCCGCATTATCTGGAAAGC	CAAGGTCCATTTCTTGGCTGT	57.9
*ABCG8*	AGCCTCCTTGCTAGATGTGAT	GTCTCTCGCACAGTCAAGTTG	57.9
*ABCG5*	ACTGCTTCTCCTACGTCCTG	CTGTAGTTGCCAATCAGTCGG	59.4
*SCD1*	TCTAGCTCCTATACCACCACCA	TCGTCTCCAACTTATCTCCTCC	60.3
*FASN*	AAGGACCTGTCTAGGTTTGATGC	TGGCTTCATAGGTGACTTCCA	60.6
*GSK3β*	CCGACTAACACCACTGGAAGCT	AGGATGGTAGCCAGAGGTGGAT	59.8
*PFKFB3*	ATTGCGGTTTTCGATGCCAC	GCCACAACTGTAGGGTCGT	58.8
*HIF1A-F*	ATCCATGTGACCATGAGGAAATG	TCGGCTAGTTAGGGTACACTTC	60.3
*GAPDH*	GGAGCGAGATCCCTCCAAAAT	GGCTGTTGTCATACTTCTCATGG	59.8

### Western blotting

Following the completion of cell treatment with T0901317 and SR9243, CSCs were lysed in a protein extraction buffer composed of 25 mm HEPES, 1% Triton X-100, 2 mM EDTA, 0.1 M NaCl, 25 mM NaF, 1 mM Sodium Orthovanadate, and protease cocktail inhibitor (Roche) on ice for 30 minutes. Cell lysates were centrifuged at 12,000 *g* for 20 minutes at 4 °C. Then, the total protein concentration of supernatants was measured using Nanodrop®. The samples were electrophoresed by 12% SDS-PAGE and transferred to PVDF membrane. A 5% skim milk solution was used to block non-specific binding sites. In this study, membranes were incubated with antibodies against ABCA1, ABCG5, ABCG8, HIF1A-F, SCD1, FASN, GSK3β, PFKFB3, and LXRα/β (Santa Cruz Biotechnology, Santa Cruz, USA) at 4 °C overnight. An appropriate secondary HRP-conjugated antibody was applied (Santa Cruz Biotechnology, Santa Cruz, USA) for 1 hour at room temperature. Finally, immunoreactive bands were detected by using ECL reagent (BioRad). The density of each band was determined using ImageJ software (version 1.4).

### Cell swelling assay

To assess the possible malfunction of ABC transporters after treatment with T0901317, CSCs were exposed to two distinct medium, including hypotonic (65% osmolality; 55.48 mM NaCl, 3.3 mM KCl, 1 mM MgCl_2_, 1.109 mM CaCl_2_, 0.9 mM MgCl_2_, 11.9 mM HEPES, and 191.2 mM Mannitol) and isotonic conditions [15]. The normal culture medium was considered an isotonic condition. In brief, 1 × 10^5^ CSCs were plated in each well of 6-well plates and incubated with 5 µM T0901317 for the next 24 h. After supernatant removal, adherent cells were washed twice with pre-warmed PBS and re-suspended in isotonic and hypotonic media. The diameter of 60 cells was recorded randomly 15, 30, and 45 min after being treated with the hypotonic condition. Finally, the average cell diameter was calculated by ImageJ software and compared to the control group.

### Measuring CSC proliferation using IF staining

To this end, we monitored the expression of Ki67, a nuclear factor, using IF. About 1 × 10^4^ CSCs were seeded in each well of 8-well Chamber Slides and treated with 5 µM T0901317 for 48 h. Then, cells were fixed with 4% paraformaldehyde solution for 10 min, washed twice with PBS, and incubated with PE-conjugated anti- Ki67 for 30 minutes at room temperature. Finally, the cells were examined using a fluorescent microscope (Olympus system, Japan).

### Clonogenic assay

The possible effect of LXR agonist and antagonist was investigated on the clonogenic capacity of CSCs after 48 h. For this purpose, 3D soft agar including 1 % gelatin and 0.1 % agarose was prepared. CSCs were treated with T0901317 (5 and 10 µM) and SR9243 (60 and 120 nM) in two separate groups. 1 × 10^3^ CSCs were mixed with 3 ml soft agar and poured in each well of 6-well plates. On day 21, CSCs colonies were counted in 10 randomly selected magnification fields.

### Migration and invasion assays

We monitored the CSCs migration rate using *in vitro* Transwell insert® and scratch assays after the modulation of LXR activity.

### Transwell insert assay

Briefly, 2 × 10^4^ CSCs were pretreated with 5 µM T09013174 in 200 µl serum-free RPMI-1640 medium and poured into polycarbonate inserts with an 8-µm pore size (SPL). In the lower chamber, we added 750 µl culture medium supplemented with 2 % FBS. After 48 h, the number of migrated CSCs was counted in random 10 fields at the bottom surface of the insert membrane stained with Giemsa.

### Invasion (scratch) assay

To this end, 1 × 10^6^ CSCs were seeded in each well of a 6-well plate and allowed to reach confluent monolayer cells and treated with 5 µM T0901317. The confluent cell layer was wounded by scratching with sterile 1000 µl disposable blue pipette tips. After 24 h, the distance between scratch edges was measured calculated using ImageJ software (NIH; Version 1.4).

### Measurement of intracellular ROS

The evaluation ROS level was done using DCFH-DA staining. In brief, SR9243-treated CSCs were incubated with 10 µg/ml of DCFH-DA for 60 minutes. Thereafter, cells were trypsinized and washed twice with PBS. Finally, cells were visualized using fluorescence microscopy. The fluorescence intensity of the treatment group was compared to the non-treated control.

### Apoptosis assay

The percent of apoptotic cells was determined after LXR inhibition/induction *via* flow cytometric analysis of Annexin V (Beyotime Biotechnology, Shanghai, China). After treatment with T0901317 and SR9243, cells were collected using an enzymatic solution. Cells were washed twice with PBS and resuspended in binding buffer (eBioscience) at 4 °C for 20 min followed by incubation in 10 µg/mL FITC-labeled Annexin V at 4 °C for 20–30 min. The percent of apoptotic CSCs were determined using the BD FACSCalibur system and FlowJo software (ver.7.6.1).

### Statistical analysis

In this study, data are expressed as mean ± SD. Statistical analysis was performed using student’s t-test and one-way ANOVA with Tukey posthoc analysis. Statistical analyses were done using GraphPad InStat software (Version 8.0.2). P values ≤ 0.05 were considered statistically significant. Statistical difference between groups is indicated by brackets as follows *p < 0.05, **p < 0.01, and ***p < 0.001.

## Results

### Flow cytometry analysis confirmed typical CSCs phenotype

**Fig. 1:**
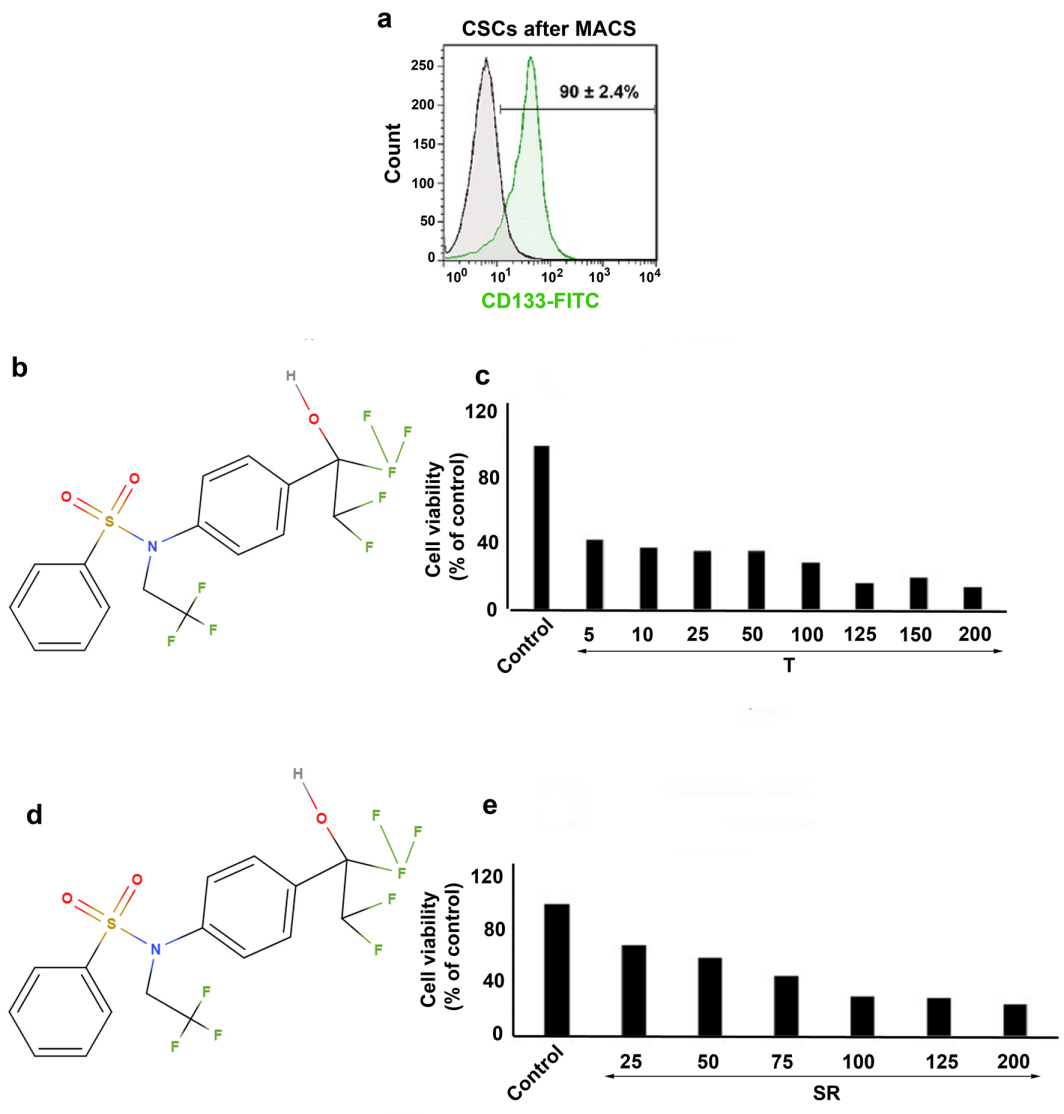
**a** CD133^+^ CSCs were enriched from human HT-29 cell line using MACS and the purity of isolated cells assessed by FITC-conjugated anti-CD133 antibody and flow cytometry analysis. **b** The chemical structure of T0901317. **c** Measuring CSCs survival after exposure to various concentrations of T0901317 (5–200 µM) using MTT assay. **d** The chemical structure of SR9243. **e** CSCs survival rate was also measured after treatment with SR9243 (25–200 nM) using MTT assay. Data confirmed a dose-dependent activity of T0901317 and SR9243 on HT-29-isolated CSCs

To enrich CSCs, we performed MACS analysis using specific antibodies against the surface CD133^+^ marker. Flow cytometry was done to confirm the purity of isolated CSCs post-MACS. According to our data, about 90 ± 2.4% of isolated cells were CD133 positive, showing that a large proportion of isolated cells exhibited a CSC-like phenotype (Fig. 1a). These data showed the successful identification and isolation of the CD133^+^ CSCs subpopulation from the HT-29 cell line.

### Inhibition and stimulation of LXR modulated CSCs viability in a dose‐dependent manner

CHOL content and lipid synthesis are pivotal factors to maintain dynamic growth in cancer cells. In support of this claim, the modulation of LXR could affect CSCs survival and growth [16]. The modulatory effect of T0901317 and SR9243 was studied on CD133^+^ CSCs using MTT assay. Data showed reduced CSCs survival rate after incubation with T0901317 (an LXR ligand), and SR9243 (an LXR antagonist) in a dose-dependent manner (Fig. 1c and e). Compared to control CSCs, ~ 2.5-fold decrease was obtained in the survival rate of CSCs exposed to 5 µM T0901317, and this value reached ~ 5.0-fold in the group treated with 200 µM T0901317. After 48-hour incubation of CD133^+^ CSCs with different doses of T0901317, the IC_50_ value was ~ 5 µM (Fig. 1c). Similar to results from T0901317-treated CSCs, we found a ~ 2.1-fold decrease in the survival rate of CSCs exposed to 25 nM SR9243 and this value reached ~ 4.5-fold in the group received 200 nM SR9243 (Fig. 1e). The results showed an IC_50_ value of ~ 69 nM for CSCs treated with SR9243. We selected IC_50_ values for subsequent analyses.

### LXR activation modulated the expression of ABC transporters

**Fig. 2:**
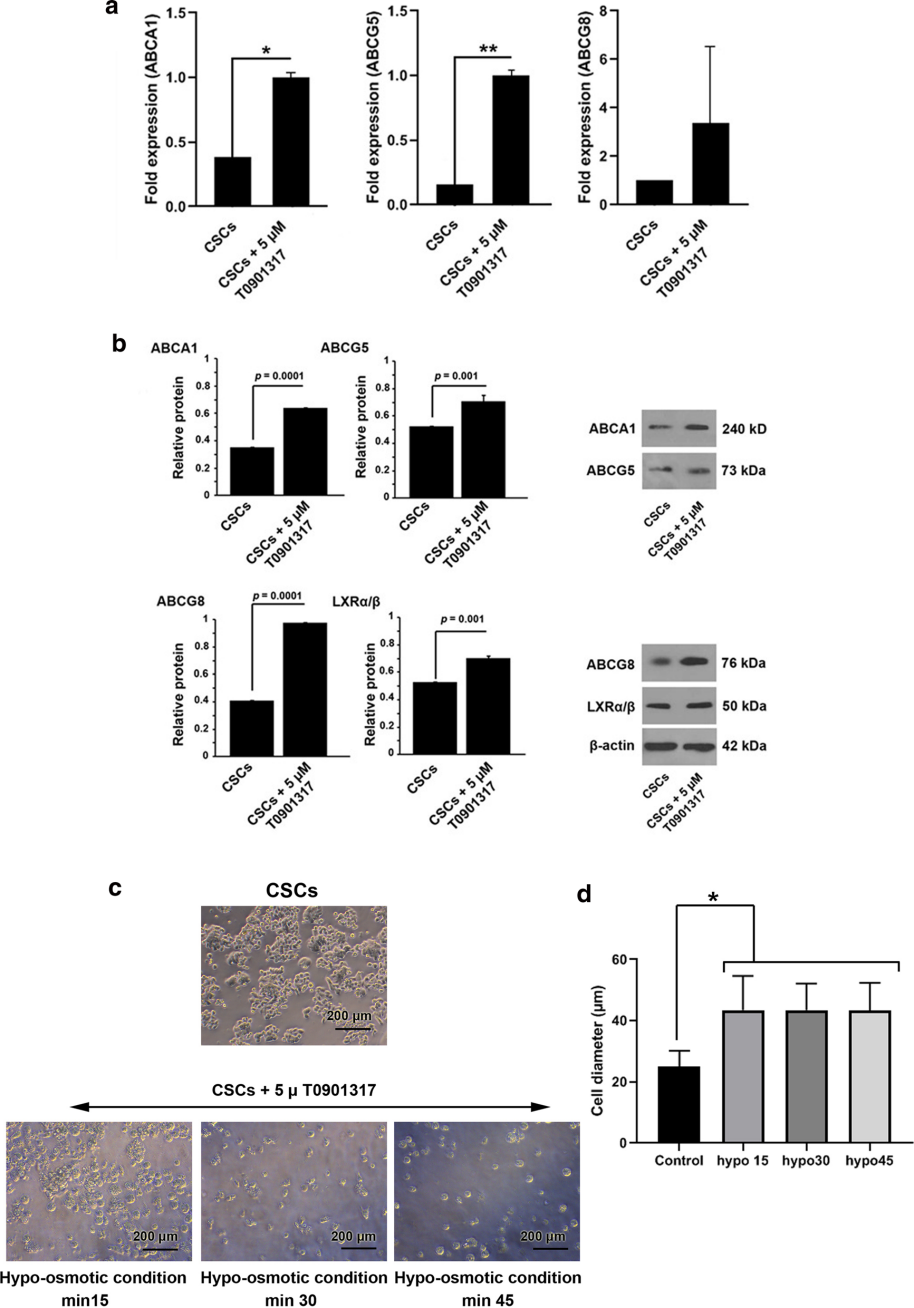
Effect of LXR agonist (T0901317) on the ABC transporter gene expression and protein levels LXRs in CSC. **a** mRNA expression levels of ABCA1, ABCG5, and ABCG8 levels were determined by RT-PCR after incubation of the treatment group with 5 µM T0901317 for 48 h. **b** Protein levels of ABCA1, ABCG5, ABCG8, and LXRs were determined by western blotting. CSCs were treated with 5 µM T0901317 for 48 h. Measuring CSCs resistance to hypo-osmotic stress after treatment with LXR agonist. **c** Changes in diameter of CSCs were recorded after exposure to 5 µM T0901317 every 15 min over 45 min (n = 60). **d** CSC diameter was increased in treated groups compared to the control group. All experiments were performed in triplicate and data were expressed as mean ± SD. Tukey posthoc analysis. *p < 0.05, **p < 0.01, and ***p < 0.001

ABC transporters participate in lipid efflux and cell resistance against osmotic pressure [17, 18]. The mRNA expression and protein levels of ABC transporters were studied by RT-qPCR and western blotting after treatment with LXR agonist T0901317. Data revealed that the incubation of CSCs with 5 µM T0901317 for 48 h increased significantly the expression of *ABCA1* (p < 0.05), *ABCG5* (p < 0.01). Despite the induction of *ABCG8* expression in comparison with the control group, the changes did not reach statistical significance (p > 0.05) (Fig. 2a). Western blotting showed a statistically significant increase of ABC proteins in the group that received 5 µM T0901317 compared to the control group (p < 0.05) (Fig. 2b). Interestingly, we found that the protein level of the LXR receptor was also increased in CSCs exposed to 5 µM T0901317 after 48 h. Commensurate with these comments, 48-hour incubation of CSCs with LXR agonist could increase ABC transporter gene expression and protein synthesis.

### Activation of LXR increased CSCs sensitivity to hypo‐osmotic stress

To decipher the possible effect of LXRs activation on CSCs resistance to hypo-osmotic stress, we measured the changes in CSCs diameter after treatment with 5 µM T0901317 in hypotonic condition. The cell size was monitored at different time points 15, 30, and 45 min. Bright-field microscopic imaging showed the changes in CSCs diameter after treatment with 5 µM T0901317 under hypo-tonic conditions (Fig. 2c). It was found that hypotonic conditions caused a more rapid cell swelling rate in T0901317-treated CSCs as compared with the control group (p < 0.05) (Fig. 2d). The mean change of cell diameter was not statistically significant in the control group over time (Fig. 2d).

### LXR activation inhibited the CSCs proliferation and clonogenic potential

**Fig. 3:**
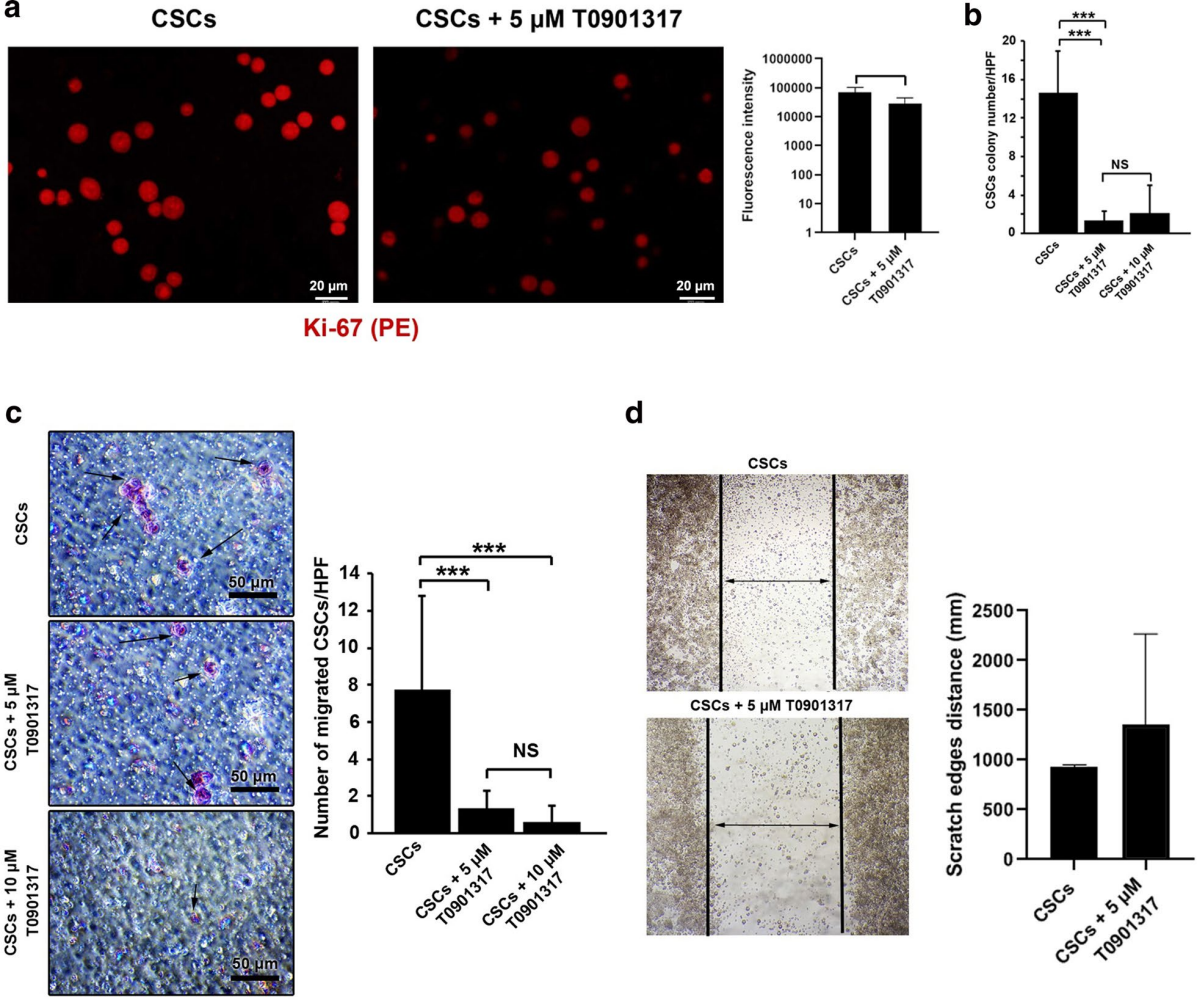
Measuring CSCs proliferation using Ki-67 factor (**a**) and clonogenic capacity after LXR activation (**b**). The fluorescence intensity of Ki-67 in CSCs with or without 5 µM T0901317 was analyzed by IF. CSCs were directly labeled using the PE-conjugated anti-Ki-67 antibody. **b** The number of colonies was decreased in after treatment with 5 and 10 µM T0901317 compared to the control group. The incubation of CSCs with T0901317 at both doses 5 and 10 µM did yield statistically significant differences (p > 0.05). Effects of LXR activation on migration and invasion CSCs after 48 h (**c** and **d**). CSCs were treated with 5 and 10 µM T0901317 and the number of migrated CSCs and invasion rates were evaluated by Transwell migration and scratch assays. **c** Transwell assays showed that the treatment of CSCs with 5 and 10 µM T0901317 decreased the number of migrated CSCs compared with the control (p < 0.001). **d** Scratch assay showed that the distance of scratch edges increased upon treatment with 5 µM T0901317 compared to the control group. However, the values did not reach statistically significant results. Student t-test and One-Way ANOVA with Tukey posthoc analysis. ^∗^p < 0.05, ^∗∗^p < 0.01, and ^∗∗∗^p < 0.001

The high level of intracellular CHOL activates LXR transcriptional and contributes to an increased CHOL efflux, reduction of CHOL influx, and synthesis [10]. IF imaging revealed that the activation of LXR in CSCs reduced the fluorescence intensity of the Ki67 antigen (a nuclear proliferation factor) compared to the control group (Fig. 3a). Taken together, these results indicated that LXR activation reduced the CSCs proliferation rate *in vitro*.

To further assess the possible effect of LXR activation on the clonogenic capacity of CSCs, cells were cultured in 3D soft agar after being treated with 5 and 10 µM T0901317. Based on data, the incubation of CSCs with the LXR activator inhibited the number of colonies after 48 h (Fig. 3b). No significant differences were observed between the number of CD133^+^ CSCs in groups 5 µM and 10 µM T0901317 groups (p > 0.05) (Fig. 3b). Taken together, these data showed that activation of the LXR receptor inhibited clonogenic capacity and malignancy.

### LXR activation reduced CSCs migration and invasion

Transwell migration assay showed that the treatment of CSCs with 5 and 10 µM T0901317 significantly suppressed the migration of CD133^+^ CSCs after 48 h compared to the non-treated CSCs (p < 0.001; Fig. 3c). However, no significant differences were observed between the number of migrated CSCs treated with 5 and 10 µM T0901317 (p > 0.05; Fig. 3a). Scratch assay showed that the treatment of CSCs with 5 µM T0901317 decreased the distance between scratch edges compared to the control CSCs but these values did not reach statistically significant differences (Fig. 3d). These results indicated that the activation of LXR suppressed CSCs migration and invasion.

### Inhibition of LXR reduced glycolysis/lipogenesis and hypoxia factor in CSCs

**Fig. 4:**
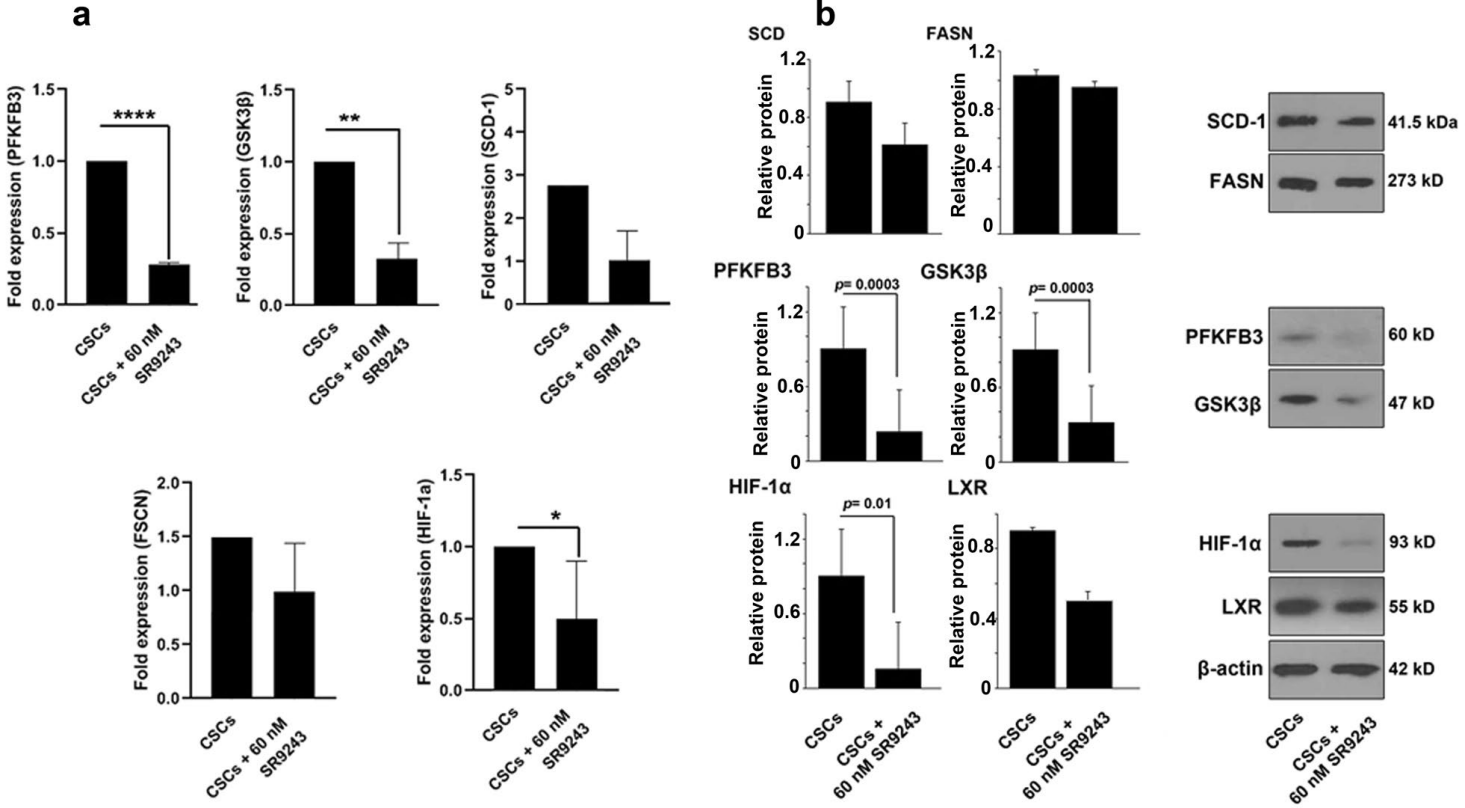
Effect of SR9243 on metabolic LXR-targets genes in CSCs. **a** mRNA expression levels of PFKFB3, GSK3β, SCD1, FASN, and HIF-1α were determined by real-time PCR after incubation with 60 nM SR9243 over 48 h. **b** Protein levels of PFKFB3, GSK3β, SCD1, FASN, HIF-1α, and LXRs were determined by western blotting in CSCs after incubation with 60 nM SR9243. All experiments were performed in triplicate and data were expressed as mean ± SD. Student t-test. *p < 0.05, **p < 0.01, and ***p < 0.001

CSCs metabolism and survival are associated with glycolysis and lipogenesis [4]. Here, we investigated whether LXR inhibition using SR9243 could alter the expression of enzymes associated with the Warburg effect (*PFKFB3* and *GSK3β*) and lipogenesis (*SCD1* and *FASN*). Here, we showed that incubation of CSCs with 60 nM SR9243 downregulated the expression of PFKFB3 (p < 0.0001), GSK3β (p < 0.01), and HIF-1α (p < 0.05) compared to non-treated CSCs. Calling attention, the expression of SCD1 and FASN were not changed in CSCs after being with 60 nM SR9243 (p > 0.05; Fig. 4a). Western blot analysis results confirmed real-time PCR results. Despite the reduction of SCD, FASN, LXR protein levels, the changes did not reach statistically significant levels (Fig. 4b). According to our data, protein levels of HIF-1α, GSK3β, PFKFB3 were significantly diminished upon treatment with 60 nM SR9243 after 48 h. These data showed that LXR inhibition could decrease the expression and protein levels of factors associated with lipolysis, hypoxia, and glycolysis.

### LXR inhibition reduced ROS content and clonogenic capacity

**Fig. 5:**
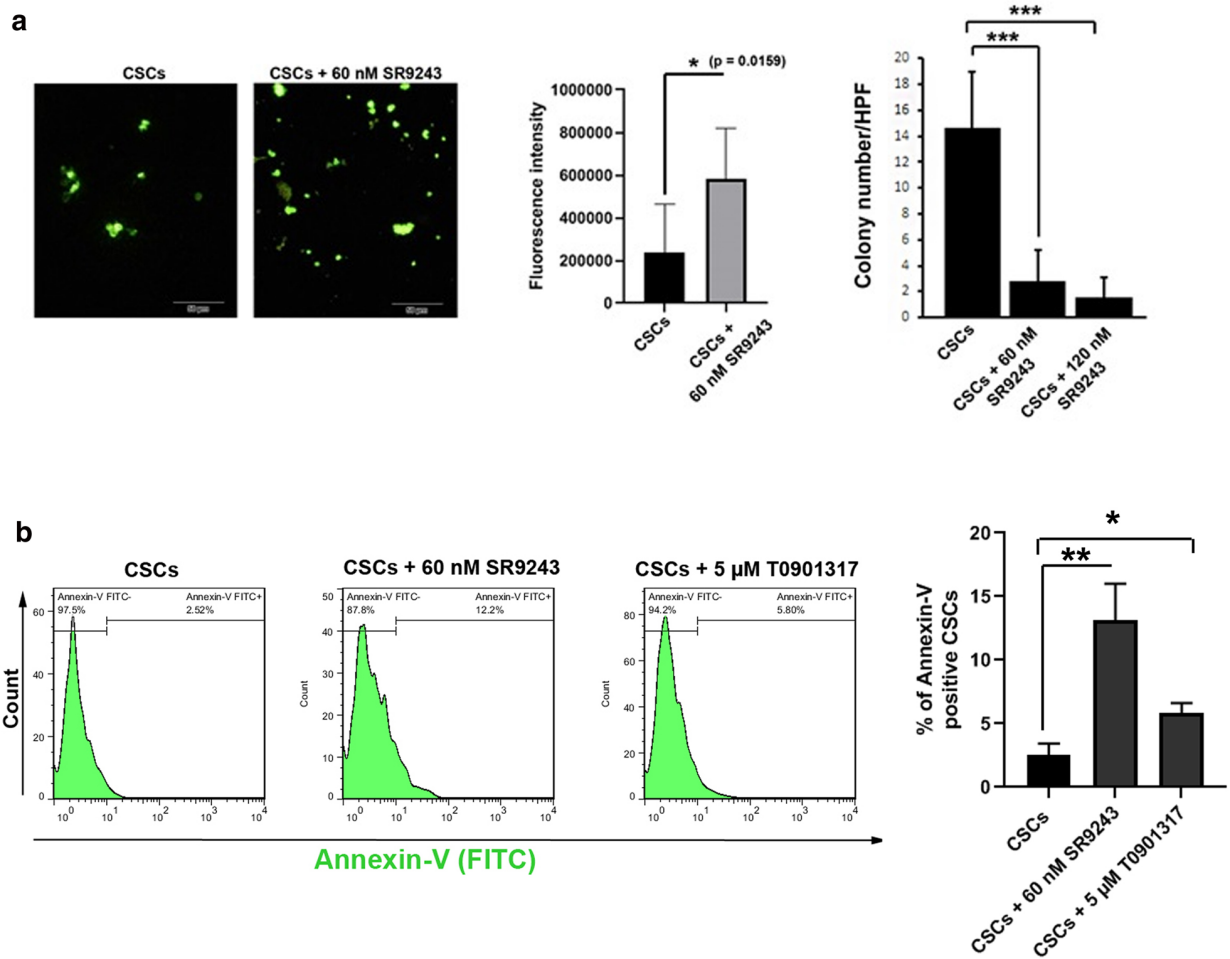
Representative image of ROS assay and colony formation capacity CSCs after being-treated with SR9243 (**a**). DCFH-DA assay showed an increase of fluorescence intensity in CSCs after exposure to 60 nM SR9243, indicating increased ROS content after LXR inhibition. The number of colony formation was calculated in in 3D soft agar after LXR inhibition. Data showed a statistically significant level between the control and treatment groups. Measuring the percent of apoptotic CSCs after-being treated with SR9243 and T0901317 (**b**). CSCs were incubated with LXR agonist (5 µM T0901317) and antagonists (60 nM SR9243) for 48 h. Student t-test and One-Way ANOVA with Tukey posthoc analysis. *p < 0.05, **p < 0.01, and ***p < 0.001

We investigated ROS content by using DCFH-DA assay after inhibition of LXR with 60 nM SR9243. Compared to the control CSCs, increased fluorescence intensity was indicated in SR9243-treated CSCs, showing that the inhibition of LXR in CSCs resulted in the accumulation of ROS after 48 h (p < 0.05; Fig. 5a). We found that 48-hour incubation of CSCs with 60 and 120 nM SR9243 profoundly diminished the colony formation capacity in a 3D soft agar compared to the non-treated control CSCs (p < 0.001; Fig. 5a). No significant differences were observed between the number of CSC colonies in groups received 60 and 120 nM SR9243 (p > 0.05; Fig. 5a).

### The modulation of LXR promoted apoptosis in CSCs

To this end, CSCs were exposed to LXR agonist (5 µM T0901317) and antagonists (60 nM SR9243) for 48 h (Fig. 5b). Based on the obtained data, the inhibition and stimulation of the LXR receptor could increase the percent of CSCs entering apoptosis after 48 h, indicated by an elevation in the number of Annexin-V positive CSCs. It seems that the inhibition of LXR had superiority to induce apoptosis compared to the group that received an LXR stimulator.

## Discussion

The main reason for the failure of CRC treatment could be explained by the presence of CSCs which possess the characteristics of self-renewal, tumorigenicity, metastasis, and metabolism heterogenicity [2]. Thus, the identification of CSCs at the molecular level will allow us to detect metastasis earlier and provide new opportunities for drug development.

The current experiment was a preliminary study to address the modulation of LXR on the dynamic growth of CSCs. Here, we showed that the activation of LXR T0901317 reduced the viability and proliferation of CSCs after 48 h. Consistent with our data, it has been shown that the stimulation of LXR could down-regulate β-catenin targets such as Bmp4, Myc, Cyclin D1, and Mmp7 participating in the survival and proliferation of colon cancer cells [19]. The effect of LXR activity on CSCs proliferation could be explained by this finding that LXR could downregulate SCARB1 gene encoding scavenger receptor B1 (SR-B1) [20]. Indeed, SR-B1 promotes CHOL uptake from the body and induces cell growth, and even promotes tumor progression or metastasis [21]. Thus, LXR activation could be resulted in SR-B1 suppression and CHOL starvation in CSCs as well as reduced their proliferation.

Based on our data, the promotion of LXR significantly induced ABC-A1/G5/G8 expression in CSCs, which confirms the results of studies conducted by Yu [22], and Repa [23]. They showed that LXR activation resulted in ABC-G5/G8 mRNAs overexpression and CHOL efflux from hepatocytes and enterocytes. In contrast, significant downregulation of the LXR and ABCB1 in the early stage of CRCs is associated with the accumulation of CHOL within tumor cells, and subsequently promotes the initial phase of CRCs development [24]. In support of our data, the stimulation of LXR in CSCs increased ABC transporters, leading to tumoricidal effects on CSCs. In hypercholesterolemia condition, the promotion of LXR followed by increased ABC efflux activity, and CHOL efflux to protect enterocytes from sterol accumulation [22, 25, 26]. Therefore, the up-regulation of ABC transporters can reduce CHOL content and tumorigenicity of CSCs.

Besides their roles in lipid metabolism, ABC transporters control the normal cell size by the release of cellular ions and loss of water *via* a regulatory volume decrease or volume-regulated anion channel [27]. In this study, we found that the activation of LXR is closely related to the function of ABC transporters in which the mean CSC diameter increased in early 15 minutes incubated in hypo-osmotic conditions. The reduced CSCs survival rate coincided with increased cell size showed that CSCs lost the capacity to resist osmotic stress. The activation of ABC transporters after LXR stimulation showed a compensatory response under hypotonic conditions. Under hypo-osmotic conditions, LXR could induce ABCA1 expression and CHOL depletion by the activation of RVD to protect CSCs from osmotic stress.

The activation of LXR seems to decrease CSCs tumorigenesis which is associated with colony formation capacity. Previously, it was shown that LXR could suppress the clonogenic capacity of CSCs via inhibiting the Hedgehog-Wnt signaling pathway which maintains CSC survival and self-renewal ability [28, 29]. Moreover, CHOL biosynthesis is essential for CSCs propagation [30], and mammosphere formation [31], that its synthesis is negatively regulated by LXR activity, and hence inhibited the CSC colony formation.

The activation of LXR seems to decrease the CSCs invasion and migration. These results could be related to the role of CHOL is an important component of membrane lipid rafts containing the signaling molecules precipitating in cell migration [16, 32, 33]. Moreover, ABCA1 modulates CHOL distribution in the membrane which affected the pathways promoting cell migration [9]. Therefore, any change in the composition of the CSC membrane could be linked to the downstream pathways which influenced the CSCs proliferation, clonogenicity, and migration. Based on our data, the promotion of LXR significantly induced apoptosis in CSCs, which could be explained based on previously published data, LXRs induced cell cycle arrest via caspase-dependent activity in HT-29 cells in a xenograft model [34].

On the other hand, in comparison with differentiated CRC cells, CD133^+^CR-CSCs favor glycolysis and lipogenesis [35], which provide potential metabolic targeting for CR-CSCs treatment. We found that inhibition of LXR activity altered the expression of PFKFB3, GSK3β, FASN, and SCD1, the critical key enzymes in the control of glycolysis and lipogenesis, and thus resulted in the reduction CSCs growth and proliferation. All of the four enzymes promote the progression of the tumor and are directly upregulated by LXRs in human somatic tumor cells [12]. Lipogenesis genes in this study, however, have shown less reduction, which may duo to the CD133^+^ CR-CSC cells have favored active glycolysis more than lipogenesis [35]. HIF-1α induces the expression of glycolytic genes [36], and we found the inhibition of LXR activity reduced HIF-1α expression. This result is inconsistent with a previous study that has reported the suppression of LXR inhibited HIF-1α and enzymes associated with glycolysis [37].

The metabolism of cancer cells and pluripotent stem cells rely on the Warburg effect to minimize ROS production [38]. Our data showed that LXR inhibition reduced Warburg effect-associated enzymes, and thus, the ROS levels were increased after LXR suppression. Notably, the inhibition of SCD1 [39], and PFKFB3 [40] can induce the ROSs generation in neoplastic cells. FASN and the low ROSs niche participate in the maintenance of stemness and the self-renewal capacity of CSCs [41] [11]. Therefore, upon treatment of CSCs with LXR antagonist SR9243, we found increased ROS content and reduced FASN which could hamper the clonogenic capacity. Besides ROS elevation, the increase of apoptosis could be related to, and PFKFB3 which later acts as an inducer of p27 degradation and cell cycle progression, hence, its silencing is found to arrest cells at G1/S and induces apoptosis [42]. In summary, SR9243 suppresses the expression of metabolic activator enzymes, and hence inhibits the cell clonogenicity and metabolism, and induces apoptosis in CD133^+^ CSCs *in vitro*. There are some limitations in this study that are associated with the lack of different CSC types from multiple cell lines. Treatment of different CSC types with LXR agonist/antagonist could help us to find possible differences in the function of LXR in several CSCs. Also, future studies must focus to find any relationship of LXR signaling with resistance mechanisms in CSCs.

## Conclusions

Here, we showed the modulation of LXR could alter the activity of CSCs *in vitro*. It seems that the LXR signaling pathway is closely correlated with different CSC bioactivities such as migration, glycolysis, lipogenesis, cell survival, and osmotic stress. However, more investigations are highly demanded to address the critical role of the LXR pathway on different CSC types.

## Data Availability

All data generated or analyzed during this study are included in this published article.

## Abbreviations

DCFH-DA: 2′, 7′-dichlorofluorescein diacetate
MTT: 3-(4, 5-Dimethylthiazol-2-yl)-2, 5-Diphenyltetrazolium Bromide
ABCs: ATP-binding cassette transporters
BSA: Bovine serum albumin
CSCs: Cancer stem cells
CHOL: Cholesterol
CRC: Colorectal cancer
DMSO: Dimethyl sulfoxide
ECM: Extracellular matrix
FASN: Fatty acid synthase
FBS: Fetal Bovine Serum
GAPDH: Glyceraldehyde-3-Phosphate Dehydrogenase
GSK3β: Glycogen synthase kinase 3 beta
HIF-1α: Hypoxia-inducible factor-1α
IF: Immunofluorescence
LXRs: Liver X receptors
MACS: Magnetic Activated Cell Sorting
PFA: Paraformaldehyde
PBS: Phosphate-buffered saline
PFKFB3: Phosphofructokinase-2/fructose-2, 6-bisphosphatase 3
ROS: Reactive oxygen species
RXRs: Retinoid X receptors
SCD1: Stearoyl-CoA desaturase 1
3D: Three-dimensional
